# The Human Nasal Microbiome: A Perspective Study During the SARS-CoV-2 Pandemic in Malta

**DOI:** 10.3390/microorganisms12122570

**Published:** 2024-12-13

**Authors:** David Pinzauti, Simon De Jaegher, Maria D’Aguanno, Manuele Biazzo

**Affiliations:** The BioArte Ltd., Life Science Park, Triq San Giljan, 3000 San Gwann, Malta; simon.de.jaegher@hotmail.fr (S.D.J.); m.daguanno@thebioarte.com (M.D.); m.biazzo@thebioarte.com (M.B.)

**Keywords:** SARS-CoV-2, COVID-19, full-length 16S, human nasal microbiome

## Abstract

The human respiratory tract is colonized by a complex microbial community that helps maintain respiratory health and plays a crucial role in defending the host from infections. Respiratory viruses have been demonstrated to alter microbiota composition, resulting in opportunistic species expansion, and increasing the disease severity and host susceptibility to bacterial co-infections. This study aims to examine the compositional differences in the nasal microbiota between SARS-CoV-2-infected and non-infected patients. We conducted Oxford Nanopore full-length 16S rRNA sequencing on nasal swabs from 94 COVID-19 negative and 85 COVID-19 positive patients collected during the SARS-CoV-2 pandemic in Malta. Our analysis identified significant alpha and beta diversity differences in the nasal microbiota composition among our study groups. We observed a trend toward decreased microbial richness and evenness in the COVID-Positive cohort with and increased abundance of common nasal opportunistic species including *Citrobacter koseri*, *Dolosigranulum pigrum*, *Haemophilus influenzae*, *Klebsiella pneumoniae*, and *Moraxella catarrhalis*. The findings from this study are in line with previously published papers identifying key alterations in the nasal microbiota composition associated with SARS-CoV-2 infection. Understanding these microbiome-driven mechanisms could present novel prognostic markers or offer new approaches for disease prevention and treatment.

## 1. Introduction

The novel severe acute respiratory syndrome coronavirus 2 (SARS-CoV-2) is the causative agent of the coronavirus disease-2019 (COVID-19) [1]. Since its appearance, the cases of COVID-19 have spread rapidly worldwide, reporting over 776 million confirmed cases and around seven million deaths as of November 2024 (https://data.who.int/dashboards/covid19, accessed on 1 November 2024). SARS-CoV-2 infections are characterized by a wide variation in clinical manifestations, ranging from asymptomatic cases to severe pneumonia and death [1,2,3,4]. Although several studies indicate that multiple factors such as age, gender, lifestyle, genetics, immune system function, and the use of antibiotics are correlated with COVID-19 severity, the mechanisms determining the infection progression remain unclear [2,3,4,5]. SARS-CoV-2 is transmitted through aerosolized droplets produced by sneezing, coughing, or simply speaking, and penetrates the host through the upper airways [1,2,6]. The upper respiratory tract (URT) is the major portal of entry and infection site for aerosol-transmitted microorganisms [1], and thus represents the first defensive line against respiratory infections [2]. Different microbial species colonize the URT forming a complex community living in a symbiotic relationship maintaining respiratory health [2,7]. The influence of the respiratory microbiome on viral infection and respiratory health has been widely studied. In fact, resident microbial communities have been shown to influence the activation and regulation of both cell-mediated and humoral immune responses, thus influencing pathogen detection and immunity, and playing a crucial role in defending the host from infections [1,3,8]. However, the presence of respiratory viruses has been demonstrated to alter the composition of the resident mucosal microbiota, resulting in diminished microbial diversity with the concurrent expansion of opportunistic pathogens, increasing the severity of the disease and host susceptibility to bacterial co-infections [1,3,8,9].

Several studies have attempted to identify and characterize the relationship between SARS-CoV-2 infections and respiratory microbiota. However, the results of these studies are relatively discordant and far from finding a consensus [1]. While many studies have shown decreased diversity in the nasal microbiome of SARS-CoV-2-infected patients versus healthy controls [6,10], other groups reported no significant changes [3,11]. Interestingly, several studies supported the idea that SARS-CoV-2 infection alters the symbiotic relationship between commensal bacteria and opportunistic pathogens that are present in the URT, resulting in an over-proliferation of pathogenic bacteria leading to the development of comorbidities [1,12].

In this work, we analyze full-length 16S rRNA sequencing data to assess compositional differences in the nasal microbiota between SARS-CoV-2-infected and non-infected patients from Malta.

## 2. Materials and Methods

### 2.1. Samples and Data Collection

This study employed a stratified random sampling methodology to ensure that the sample population accurately reflected the diverse characteristics of the larger population. The population was initially divided into distinct subgroups, or “strata”, based on gender (male and female) and COVID status criteria (negative and positive, based on RT-qPCR results). The stratification ensured that both gender and COVID status were considered, allowing for the targeted analysis of each subgroup’s characteristics and outcomes. Once the strata were established, a random sample was drawn from each group to ensure representation. The random selection from each stratum minimized sampling bias and ensured that all subgroups were adequately represented in the overall sample. This method enhanced the reliability of the findings, as it allowed for a comprehensive understanding of the population [13].

Nasal swab specimens were collected with Yocon Viral Transport Medium (Yocon Biology, Beijing, China) during the SARS-CoV-2 pandemic in Malta (from February 2021 to August 2022) (Table S1) and processed the day of sampling, testing for SARS-CoV-2 presence at The BioArte Ltd. (Malta Life Science Park, San Gwann, Malta). Routine diagnostic testing was performed using a Viasure Viral SARS-CoV-2 RT-qPCR kit (CerTest Biotec, Zaragoza, Spain) targeting the ORF1ab and Nucleocapsid (N) viral proteins. The procedure was performed according to the kit manufacturer’s guidelines. A cycle threshold (Ct) cut-off value of 40 was used for the classification of the samples. In the case of RT-qPCR positivity (Ct < 40), samples were labelled as “COVID-Positive” and screened for potential SARS-CoV-2 variants. A SARS-CoV-2 PCR tiling sequencing approach was adopted [14] using the COVID Mini kit (Oxford Nanopore Technologies, ONT, Oxford, UK). Data analysis was carried out using the wf-artic Nextflow workflow (https://github.com/epi2me-labs/wf-artic, accessed on 1 November 2024) with default parameters. The positive samples were further divided into three subgroups based on the measured Ct values: samples with reported Ct < 25 cycles were labelled as “Low Ct”, while samples with Ct ≥ 32 were labelled as “High Ct”. The samples with Ct values in the range between 32 and 25 cycles were labelled as “Middle Ct”. The specimens with no detectable SARS-CoV-2 (Ct ≥ 40) were labelled as “COVID-Negative”.

### 2.2. Full-Length 16S rRNA Sequencing

Total DNA from the nasal swabs was isolated using the MagMax Microbiome Ultra Nucleic Acid Isolation Kit (Applied Biosystems, Carlsbad, CA, USA), in combination with a KingFisher Flex Purification System (Thermo Fisher Scientific, Waltham, MA, USA). The samples were lysed using the homogenizer MP FastPre-24 5G (MP Biomedical, Irvine, CA, USA) relying on a bead-beating approach (mechanical lysis). DNA extraction was performed according to the manufacturer’s instruction. Isolated DNA was quantified with the Qubit 4 Fluorometer (Thermo Fisher Scientific) using the dsDNA high sensitivity (HS) kit.

The full-length 16S rRNA gene (~1500 bp in length) was amplified with the primers 27f (5′-TTTCTGTTGGTGCTGATATTGC-AGRGTTYGATYMTGGCTCAG-3′) and 1492r (5′-ACTTGCCTGTCGCTCTATCTTC-CGGTTACCTTGTTACGACTT-3′) as previously described [15], introducing a few modifications. The PCR reaction was carried out in a 25 µL total volume containing 12.5 µL of LongAmp Taq 2x Master Mix (New England Biolabs, Ipswich, MA, USA), the primers (400 nM), and 8.5 µL of template DNA. The reaction was run in a T100 thermal cycler (BioRad, Hercules, CA, USA) using the following program: initial denaturation at 95 °C for 4 min; followed by 30 cycles at 95 °C for 20 s, 51 °C for 30 s and 65 °C for 4 min; and a final extension 65 °C (5 min). The PCR products were checked on a 2% agarose gel and cleaned with Agencourt AMPure XP beads (Beckman Coulter, Indianapolis, IN, USA). Briefly, 0.6x AMPure beads were added to the reaction, incubating for 5 min at room temperature. Beads were pelleted using a magnetic rack (NimaGen, Nijmegen, Netherlands), washed twice with freshly prepared 70% ethanol, and resuspended in 15 µL of nuclease-free water. After 10 min of incubation at room temperature, beads were pelleted again in the magnetic rack and the eluate was collected. Yields and purity values were measured using a NanoDrop 8000 spectrophotometer (Thermo Fisher Scientific).

Each sample was associated with a unique molecular barcode sequence enabling sample multiplexing. Molecular barcodes were added in a second PCR reaction, using a modified version of the “PCR Barcoding Expansion 1–96 kit” (Oxford Nanopore Technologies). A customized panel of 5′-phosphorylated primers was used to skip the End-Prep and dA tailing reactions (ONT), reducing the time and overall costs. Barcoding PCR was carried out in final reaction volume of 25 µL: 12.5 µL of LongAmp Taq 2x Master Mix (New England Biolabs), 0.5 µL of primer mix (10 µM), and 100 fmol of 16S amplicons from the previous reaction. The reaction was run in a T100 thermal cycler (BioRad) with the initial denaturation at 95 °C for 3 min; 12 cycles of 95 °C for 15 s, 62 °C for 15 s, and 65 °C for 4 min; and a final extension at 65 °C for 15 min. The PCR products were checked on a 2% agarose gel, cleaned with 0.6x AMPure XP beads (as previously described), and quantified with a NanoDrop 8000 spectrophotometer.

The barcoded amplicons were pooled together in a 30 µL volume containing 500 ng DNA. Sequencing adapters were ligated using the ligation sequencing kit SQK-LSK114 (ONT). In short, 12.5 µL of Ligation Buffer (LNB), 5 µL of Quick T4 Ligase, and 2.5 µL of Ligation Adapter (LA) were added to the 30 µL pool. The reaction was incubated at room temperature for 10 min. AMPure XP beads (0.4x) were added to the reaction and incubated for 5 min. Beads were pelleted on a magnetic rack and washed twice with 250 µL of Short Fragment Buffer (SFB). The bead pellet was then resuspended in 15 µL of elution buffer, incubated for 10 min at room temperature, and pelleted again in the magnetic rack. After pelleting, the eluate was carefully transferred into a clean Eppendorf DNA LoBind tube. Flow cell priming and library loading were performed according to the manufacturer’s instructions: 10 fmol of DNA library was gently loaded in a dropwise manner onto a R10.4.1 flow cell (ONT). The sequencing run was performed using a GridION Mk1b device (ONT), enabling real-time super-accurate basecalling and live demultiplexing (using default parameters).

### 2.3. Data Analysis Workflow

Oxford Nanopore native pod5 files were basecalled and demultiplexed live using Guppy v. 6.1.5 (super accurate mode, quality threshold > 10); readouts were analyzed using NanoPlot v. 1.41.0 [16]. Basecalled fastQ files were trimmed using cutadapt v. 3.5 [17] removing sequencing adapters and synthetic extremities, and then filtered with dada2 v. 1.28.0 R package [18]: reads between 1200 and 1800 nucleotides in length [19] and a maximum expected error rate of 45 (maxEE = 45) were selected, discarding low-quality reads. Chimeric reads and putative chimeras were filtered out using minimap2 v. 2.16 [20], performing an all-vs-all read overlap (-x ava-ont preset, maximal distance between the seed of 500) and yacrd v. 1.0.0 [21] run with default parameters. Finally, taxonomy was inferred using emu v. 3.4.4 [22] and the Genome Taxonomy Database (GTDB) reference database [23]. The GTDB database (release v. 207) was downloaded from the official repository (https://data.gtdb.ecogenomic.org/releases/, accessed on 1 March 2023) and manually curated using the tool RESCRIPt v. 2021.11 [24]. In short, reference 16S rRNA genes were filtered, removing i) sequences containing 5 or more ambiguous bases and any homopolymers that were 8 or more bases in length (culling low-quality), and ii) sequences shorter than 1200 nucleotides in length. A final dereplication step was performed, removing redundant sequences, and keeping the unique features (--p-mode ‘uniq’ parameter) (https://github.com/bokulich-lab/RESCRIPt), accessed on 1 March 2023). The resulting tsv file, containing identified microbial species and counts, was exported to R for statistical analysis.

### 2.4. Statistical Analysis

Statistical analyses were carried out in R v. 4.3.1 [25], embedded in RStudio v. 524 [26]. We identified and removed presumed contaminants based on their presence in negative control samples using the prune_taxa function from the phyloseq R package v. 1.44 [27]. Samples with less than 5000 mapped reads were also removed. Alpha diversity indexes were calculated using the phyloseq tool, evaluating the Shannon and Simpson diversity indexes and the number of unique species detected. The Wilcoxon rank sum test was implemented to identify significant changes in alpha diversity. Following, sample counts were centered log-ratio (CLR)-transformed using the microbiome R packages v. 1.22 [28], evaluating the beta diversity (Aitchison distance dissimilarity) between the COVID-Negative and -Positive groups. Principal component analysis (PCA) and permutational multivariate analysis of variance (PERMANOVA) based on Euclidean distances (*adonis2* function, *vegan* R package v. 2.6.4) [29] were used to evaluate the associations between our study groups and the nasal microbiome composition. Differential abundance analysis (DAA) was performed using the R package *ANCOM-BC* v. 2.4.0 [30]. A false discovery rate (FDR) was implemented to adjust the computed *p*-values (p_adj_method=“fdr”), where a maximum number of iterations of 300 was selected (max_iter = 300). Finally, a generalized linear model (GLM) was implemented in our study to predict the COVID-Negative vs. -Positive outcome. The first three PCs were isolated from the centered log-ratio-transformed abundance and used as predictors in a GLM approach (principal component regression). A penalized maximum likelihood estimation model was used (*lrm* function, *rms* v. 6.7 package [31]), enabling bootstrap resampling (B = 300) to obtain an out-of-sample estimate of model performance.

## 3. Results

### 3.1. Study Population

One hundred seventy-nine patients (*n* = 179) were enrolled in the present study. Participants were classified as COVID-Negative (*n* = 94, 52.51%) and COVID-Positive (*n* = 85, 47.49%), according to the ViaSure Viral SARS-CoV-2 RT-qPCR kit (CerTest Biotec) results. Our study exhibited similar COVID-Negative and -Positive sample distribution (Table 1).

In the COVID-Positive cohort, 50 samples were classified as the SARS-CoV-2 Omicron variant, 11 as the Delta variant, 5 as the Alpha variant, and 1 as the Eta variant (https://stacks.cdc.gov/view/cdc/105817, accessed on 1 November 2024). The remaining 18 out of the 85 COVID-Positive samples (21.17%) failed the sequencing run or did not generate enough data to be classified into a viral lineage (e.g., high error index or gaps in the consensus sequence).

### 3.2. Sequence Data Quality and Filtering

The full-length 16S rRNA amplicon sequencing generated a total of 30 million reads with a mean read length of 995.8 bases in length (N50 read length–1501 bp) and a mean quality value of 17.8. The sequencing reads below the quality threshold, chimeras, and reads shorter than 1200 bp or longer than 1800 bp were discarded. The reads that passed the quality control were classified using emu [22] inferring taxonomy. In total, 152 samples (COVID-Negative, *n* = 78 and COVID-Positive, *n* = 74) passed the quality thresholds. The computed mean read length, mean quality, and N50 read length were 1449.9 bp, 18.6, and 1460 bp, respectively. We obtained an average of 51,000 sequencing reads from the 152 samples included in this analysis. A comparable sequencing depth was achieved even though the COVID-Positive samples showed smaller sequencing depths (Figure S1).

### 3.3. Richness, Evenness, and Diversity Metrics

To ensure that the differences observed between the COVID-Negative and COVID-Positive groups were not influenced by potential confounders, we implemented the Chi-square test to assess whether demographic characteristics (age, gender, and ethnicity) differed significantly between the groups. A statistically significant difference was observed for ethnicity (*p* = 0.020), while no significant differences were observed for age (*p* = 0.835) or gender (*p* = 0.141). Given the significant association with ethnicity, we included it as a covariate in alpha and beta diversity analyses to account for its potential confounding effect.

**Figure 1:**
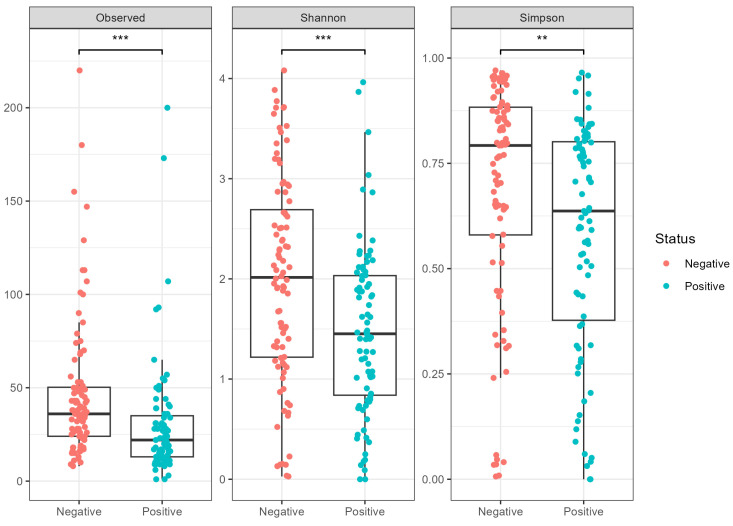
Alpha diversity. Species-level differences in the nasal microbiota composition between COVID-Negative (red) and COVID-Positive (blue) patients. Asterisks refer to the *p*-value, where ** *p*-value < 0.01 and *** *p*-value < 0.001.

Significant differences in alpha diversity among COVID-Negative and COVID-Positive patients were observed (Figure 1), detecting significantly lower Shannon/Simpson diversity and evenness in the SARS-CoV-2-infected patients compared to the uninfected samples. No significant differences were observed when ethnicity was included as a confounding factor.

**Figure 2:**
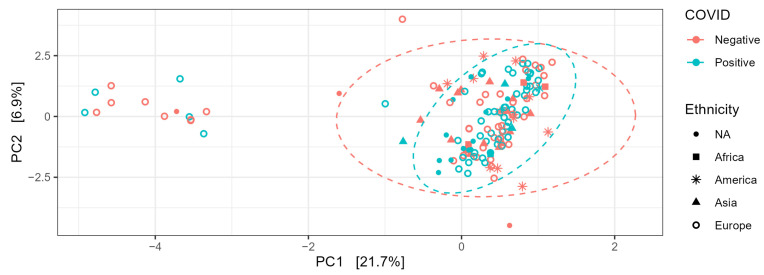
Beta diversity. Principal component analysis (PCA) plot based on Euclidean distances among COVID-Negative (red) and COVID-Positive (blue) groups (Aitchison distance dissimilarity ordination). Samples were shaped by corresponding ethnicity. Samples with unknown or missing ethnicity are represented by a hollow circle (NA).

Euclidean distance ordination was performed to assess the differences in beta diversity using principal component analysis (PCA) at the species-level taxonomic rank. Statistical analysis was implemented to determine whether there was significant clustering among our study group. Although there was a large degree of overlap in the 95% confidence ellipses, we observed significant clustering in the microbial composition between the COVID-Negative and COVID-Positive groups (*p* = 0.028, R^2^ = 0.011, PERMANOVA) (Figure 2). Significant differences in the beta diversity were also observed when ethnicity was accounted for (*p* = 0.012, R^2^ = 0.041, PERMANOVA).

**Figure 3:**
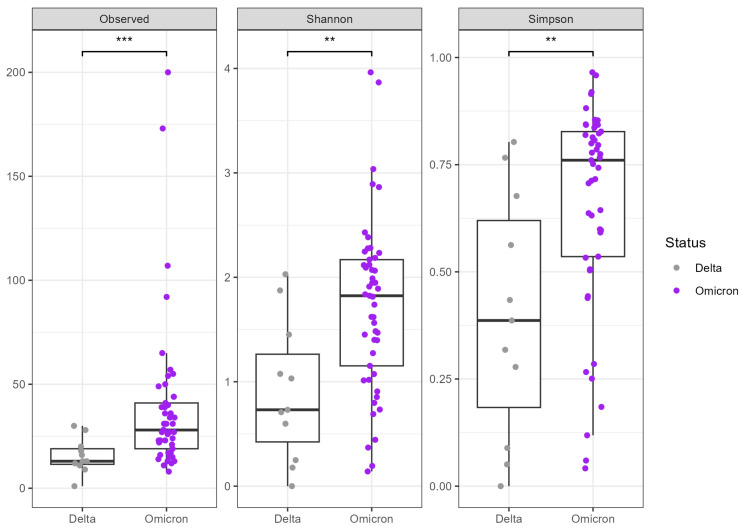
Alpha diversity relating to the SARS-CoV-2 variants. The figure represents the measured alpha diversity between the SARS-CoV-2 Delta (grey) and Omicron (purple) variants. Computed *p*-values are represented using asterisks, where ** *p*-value < 0.01 and *** *p*-value < 0.001.

Among the COVID-Positive samples, we observed significant differences in the alpha diversity between the SARS-CoV-2 Omicron and Delta variants (Wilcoxon rank-sum test *p* = 0.003 Simpson, *p* = 0.002 Shannon) (Figure 3), showing lower richness and evenness in the Delta-infected patients. We did not observe significative differences in the nasal microbiota compositions related to Ct values (Figure S2), although there was a trend towards a lower mean diversity and evenness in samples with higher SARS-CoV-2 viral loads (measured Ct values < 25).

### 3.4. Nasal Microbiome Composition

The sequencing reads were classified into 1.069 bacterial species representing 365 genera. Microbial communities in both the COVID-Negative and COVID-Positive groups were dominated by *Firmicutes* (65.5% and 64.4%, respectively), *Actinobacteria* (22.7% and 19.7%, respectively) and *Proteobacteria* (8.6% and 14.6%, respectively).

**Figure 4:**
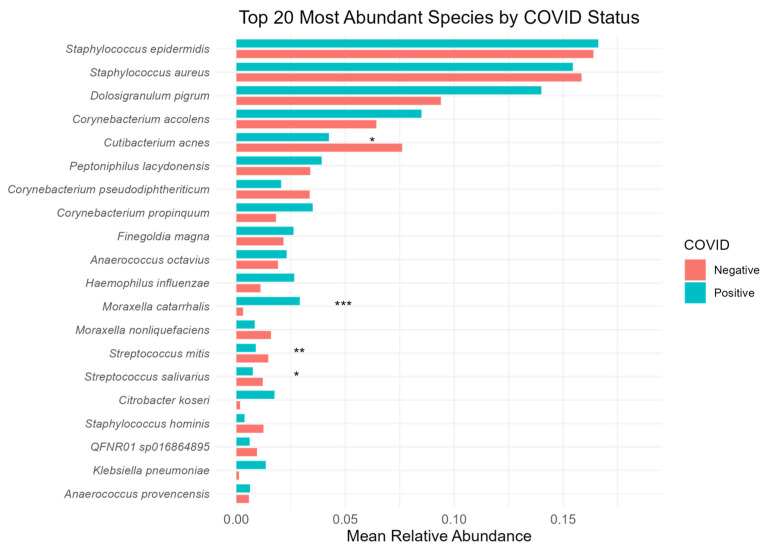
Species-level nasal microbiota composition. Bar plot showing the top 20 most abundant bacterial species on average. Species names are listed on the Y-axis, while mean relative abundance are on the X-axis. COVID-Negative samples are represented in red while COVID-Positive are in blue. Wilcoxon rank-sum test-computed *p*-values are represented using asterisks, where * *p*-value < 0.05, ** *p*-value < 0.01, and *** *p*-value < 0.001.

Eight bacterial genera accounted for more than 80% of the sequencing reads identified in our cohort of samples: *Staphylococcus* (34.6% and 33.4%), *Corynebacterium* (12.5% and 14.5%), *Dolosigranulum* (9.4% and 14.0%), *Cutibacterium* (8.1% and 4.6%), *Streptococcus* (7.3% and 4.5%), *Peptoniphilus* (3.5% and 4.0%), *Anaerococcus* (3.2% and 3.4%), and *Moraxella* (1.9% and 4.5%). Two members of the *Staphylococcus* genus, the opportunistic pathogen *Staphylococcus aureus* and *Staphylococcus epidermidis*, being the most abundant genera on average in both the COVID-Negative and COVID-Positive samples (34.6% and 33.4%, respectively). At the species level, the COVID-Positive samples displayed a higher mean relative abundance of common nasal pathobionts and opportunistic pathogens, *Citrobacter koseri* (0.2% and 1.7%), *Dolosigranulum pigrum* (9.4% and 14.0%), *Haemophilus influenzae* (1.1% and 2.7%), *Klebsiella pneumoniae* (0.1% and 1.3%), and *Moraxella catarrhalis* (0.3% and 2.9%) (Figure 4). Statistically significant differences (Wilcoxon rank-sum test) in species abundance were observed for the *C. acnes* (*p* = 0.0180), *M. catarrhalis* (*p* = 0.0019), *S. mitis* (*p* = 0.0077), and *S. salivarius* (*p* = 0.0138) species. The species *Moraxella catarrhalis* was found to be significantly and differentially abundant with ANCOM-BC analysis (Q-value = 0.0178).

**Figure 5:**
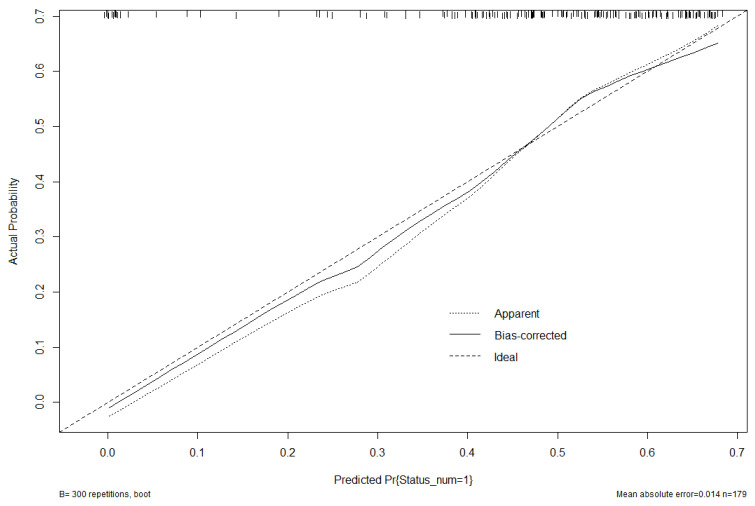
Generalized linear model predictive model. Calibration curve of a logistic regression predictive model. The X-axis represents the predicted probabilities according to the model, while the Y-axis shows the observed proportion of matching. The model overall appears to be discreetly calibrated as the Bias-Corrected line closely follows the Ideal line (mean absolute error 1.4%, 90th percentile absolute error 2.3%). Around 0.3, the predicted probability of the Bias-Corrected line is above the Ideal, suggesting overprediction, while around 0.6, the Bias-Corrected line is below the Ideal line, suggesting underprediction at higher predicted probabilities.

The GLM predictive model achieved a good overall calibration (calibration intercept of −0.0082) with slight overfitting (calibration slope of 1.119). The model showed moderate discrimination ability (Somers’ Dxy score of 0.377 and C-Statistic score of 0.688) and acceptable accuracy as reflected by a Brier score of 0.210. A mean absolute error rate of 1.4% (90th percentile absolute error 2.3%) was measured, indicating relatively low prediction error (Figure 5).

## 4. Discussion

In the present study, we used the full-length 16S rRNA sequencing approach to evaluate differences in the nasal microbiota composition associated with SARS-CoV-2 infection. Our results are consistent with previously published studies [6,9,32] identifying diminished microbial diversity in COVID-19 positive patients with a concurrent increase in the mean relative abundance of putative nasal pathobionts and opportunistic species.

We identified significant alpha and beta diversity differences in the COVID-Positive individuals versus the -Negative individuals. However, we observed a significant overlap in the confidence ellipses (Figure 2), suggesting that the magnitude of differences in the microbiota composition among our study groups is relatively small (although significant, *p* = 0.032) [3]. The high relative abundance of *Staphylococcus* spp. among our study groups (34% relative abundance on average) is consistent with the literature describing it as a “core” member of the nasal microbiota community [3,33]. The presence of differentially abundant *Moraxella catarrhalis* (ANCOM-BC Q-value = 0.0178) [1,5,33] and a trend towards a higher mean relative abundance of common nasal opportunistic species, which was observed in the COVID-Positive cohort (Figure 4), potentially highlights an increase in the prevalence of pathobionts and opportunistic pathogens during SARS-CoV-2 infection [1,3]. Interestingly, a higher abundance of *Cutibacterium accolens* and *Dolosigranulum pigrum* were measured in the COVID-Positive cohort. *C. accolens* and *D. pigrum* are two common inhabitants of the human nares and are generally considered promoters of a healthy respiratory status, playing a protective role against viral and bacterial infections [2,7,8,33]. However, higher *D. pigrum* abundance was identified in several other viral infections such as metapneumovirus, influenza A and B viruses, RSVs, and HRVs [1,5], while, on the contrary, a reduction in *C. accolens* was detected in the SARS-CoV-2-infected nasopharyngeal microbiome [6].

Since the beginning of the pandemic, several SARS-CoV-2 variants have emerged and spread globally. These variants were characterized by different clinical outcomes and immunological responses, resulting in varying transmissibility and disease severity, which might influence the respiratory microbiota composition. In the present study, we identified significative differences in the nasal microbiota composition between the Delta- and Omicron-infected patients, even though a distinct separation along the PCA axes was not observed. The results are in line with the current literature: Nath et al. [12] characterized differences in URT microbiota related to SARS-CoV-2 variants suggesting that “the extent of dysbiosis in the URT of Omicron-infected patients is lesser than in the Delta-infected patients” [12]. Interestingly, we did not observe any significative differences in nasal microbiota depending on the Ct values, suggesting that the viral load was not influencing the microbiota composition [3,9,23,33].

The implemented GLM model suggested that we can predict SARS-CoV-2-infected patients from the negative controls with moderate accuracy (C-statistic score of 0.688 and Somers’ Dxy score of 0.377), using bacterial species as predictors. These results emphasize the potential of microbiome data for distinguishing between healthy and diseased states. However, further improvements are necessary to refine the prediction model.

Our study had several limitations. First, the samples were de-identified, making it impossible to adjust the potential confounders that may have influenced the nasal microbiota such as medications, medical interventions, antibiotic usage, and the lack of medical evaluation. We acknowledge a lack of inclusion of in-depth medical evaluation during the COVID-19 testing, resulting in the impossibility to determine if or how many of the COVID-Negative samples were representative of the nasal microbiota composition of healthy subjects. In the present study, we used nasal swabs to evaluate the relationship between SARS-CoV-2 infection and nasal microbiome composition. However, reference RT-qPCR diagnostic tests are based on nasopharyngeal swabs [34], allowing for the study of the nasopharyngeal microbiome. Because the respiratory microbiota is shaped by different anatomical niches of the respiratory tract (niche specificity) [1,7], comparing the results we achieved in the present study against the literature represents an important limitation. However, as pointed out by Rhoades et al. [33], there is a clear need to understand the interaction between the host and nasal microbiome during SARS-CoV-2 infections as the nasal cavity represents the first infection site.

The results of this study present several key alterations in the nasal microbiota composition associated with SARS-CoV-2 infection. Alteration in the nasal microbiome and the subsequent expansion and colonization of pathobionts can result in a higher risk of developing severe outcomes [21]. This study adds to the current literature, emphasizing the value of microbiome data in relation to respiratory infection. However, further work is necessary to determine whether functional characteristics of the nasal microbiome are associated with respiratory viral infection and adverse patient outcomes. Understanding these microbiome-driven mechanisms could present novel prognostic markers or offer new approaches to disease prevention and treatment. Hence, it is of immense importance to maintain nasal and oral health during COVID-19 infection to prevent severe outcomes [12,33,35].

## Figures and Tables

**Table 1:** Characteristics of the study population.

	COVID-Negative (*n* = 94)		COVID-Positive (*n* = 85)	
	N. (Median)	%	N. (Median)	%
Age group:				
0–14	6 (4)	6.2%	6 (7)	7.1%
15–44	54 (36)	57.5%	42 (33)	49.4%
45–64	16 (55)	17.0%	9 (47.5)	10.6%
>65	18 (68.5)	19.2%	28 (69)	32.9%
Gender:				
Female	40	42.6%	38	44.7%
Male	49	52.1%	39	45.9%
NAs	5	5.3%	8	9.4%
Ethnicity:				
African	3	3.2%	1	1.2%
American	14	14.9%	1	1.2%
Asian	17	18.1%	3	3.5%
European	50	53.2%	57	67.1%
NAs	10	10.6%	23	27%

Characteristics of the study participants based on age, gender, and nationality. NA: unknown, lack of information.

## Data Availability

The dataset supporting the conclusions of this article is available in the European Nucleotide Archive (ENA) repository, PRJEB75547.

## Supplementary Materials

The following supporting information can be downloaded at: https://www.mdpi.com/article/10.3390/microorganisms12122570/s1, Table S1: BIOMEVAC metadata sheet; Figure S1: Oxford Nanopore sequencing depth; Figure S2: Alpha diversity related to RT-qPCR Ct values.
