# Supplementary figures and images for: The Human Nasal Microbiome: A Perspective Study During the SARS-CoV-2 Pandemic in Malta
Source: Microorganisms. 2024 Dec 13;12(12):2570. doi: 10.3390/microorganisms12122570 (PMC11679825; doi:10.3390/microorganisms12122570)

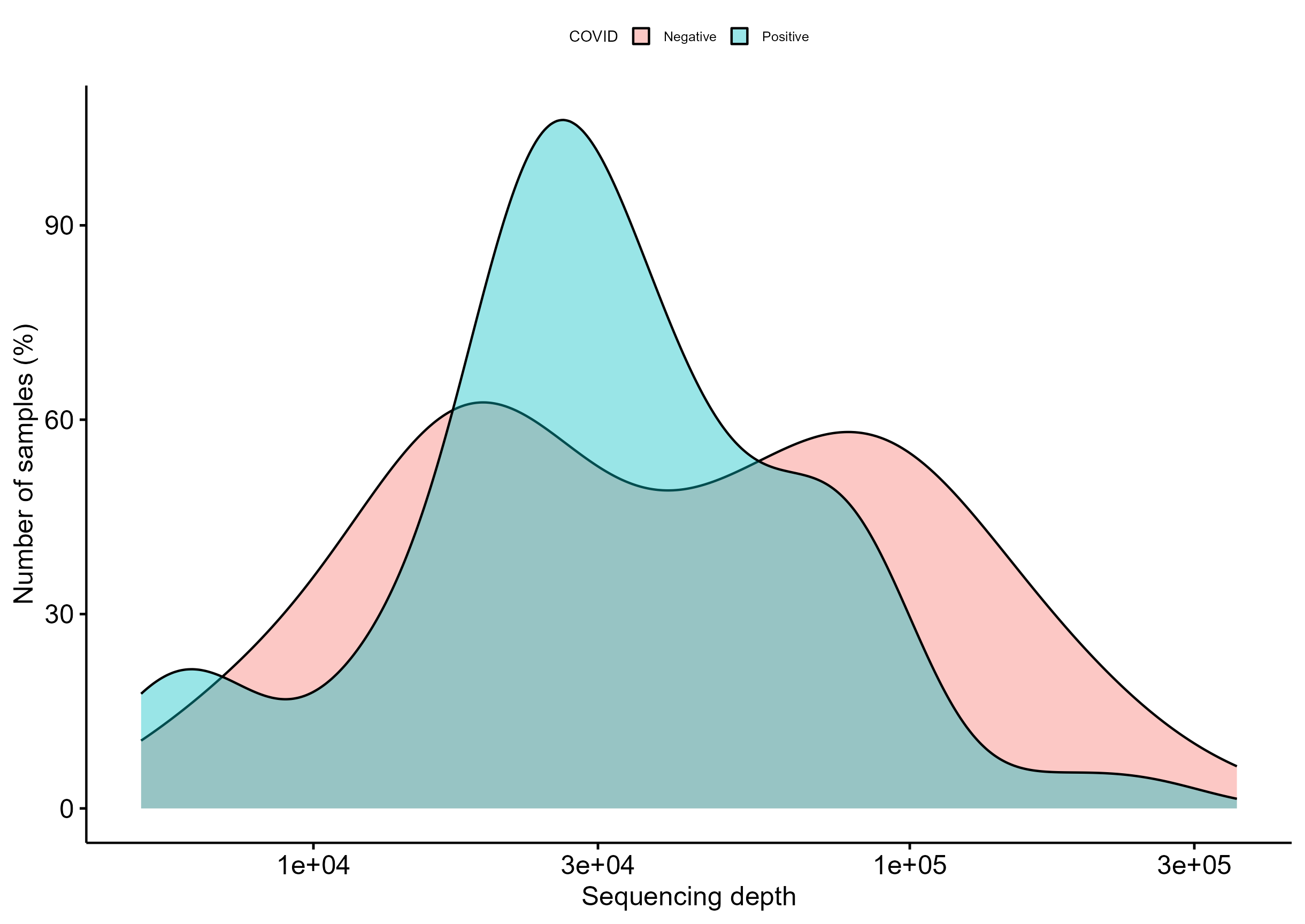

Supplement: Supplementary file 1 [file microorganisms-12-02570-s001.zip › Figure S1.png]

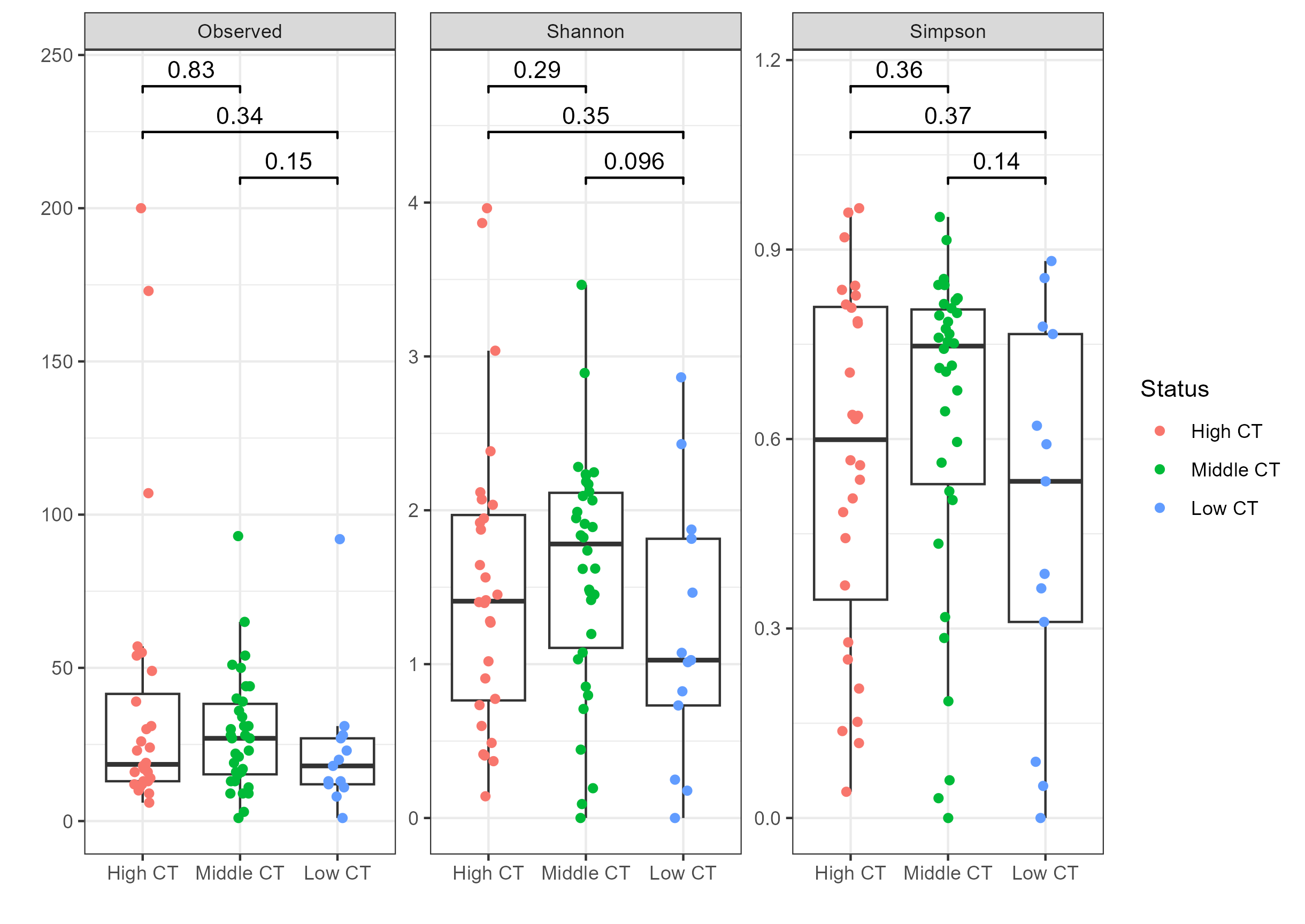

Supplement: Supplementary file 1 [file microorganisms-12-02570-s001.zip › Figure S2.png]
